# Method for combined biometric and chemical analysis of human fingerprints

**DOI:** 10.1007/s12127-014-0148-6

**Published:** 2014-03-15

**Authors:** Jessica L. Staymates, Shahram Orandi, Matthew E. Staymates, Greg Gillen

**Affiliations:** 1 Materials Measurement Science Division, National Institute of Standards and Technology (NIST), Mailstop 8371, 100 Bureau Drive, Gaithersburg, MD 20899 USA; 2 Information Access Division, National Institute of Standards and Technology (NIST), Mailstop 8940, 100 Bureau Drive, Gaithersburg, MD 20899 USA

**Keywords:** Trace detection, Biometrics, Fingerprints, Explosives, Narcotics, Ion mobility spectrometry

## Abstract

This paper describes a method for combining direct chemical analysis of latent fingerprints with subsequent biometric analysis within a single sample. The method described here uses ion mobility spectrometry (IMS) as a chemical detection method for explosives and narcotics trace contamination. A collection swab coated with a high-temperature adhesive has been developed to lift latent fingerprints from various surfaces. The swab is then directly inserted into an IMS instrument for a quick chemical analysis. After the IMS analysis, the lifted print remains intact for subsequent biometric scanning and analysis using matching algorithms. Several samples of explosive-laden fingerprints were successfully lifted and the explosives detected with IMS. Following explosive detection, the lifted fingerprints remained of sufficient quality for positive match scores using a prepared gallery consisting of 60 fingerprints. Based on our results (*n* = 1200), there was no significant decrease in the quality of the lifted print post IMS analysis. In fact, for a small subset of lifted prints, the quality was improved after IMS analysis. The described method can be readily applied to domestic criminal investigations, transportation security, terrorist and bombing threats, and military in-theatre settings.

## Introduction

Friction ridge skin impressions, or latent fingerprints, are an extremely important piece of trace evidence often discovered at the scene of a crime. Each person has unique fingerprints, and people can unintentionally leave detailed impressions of these friction ridges specific to their fingers on the objects they touch. Such latent fingerprints are often developed, photographed, and collected at the crime scene, and the images are later compared to known prints for a possible identification match [10]. Latent fingerprints are typically composed of a mixture of sebum and sweat excretions, and can also be contaminated with substances that a person has handled such as narcotics or explosives [14, 8, 3]. The ability to screen for these substances in a latent fingerprint is highly beneficial for placing an individual at a specific scene, and to determine what contraband that person may have come into recent contact with Wynn et al. [14], Ng et al. [8], Day et al. [3], Hazarika et al. [6], Chen et al. [2], Bhargava and Perlman [1] and Mou and Rabalais [7].

This paper describes the development of a method for combined chemical and biometric analysis of lifted fingerprints. The ideal characteristics for such a method include a low-cost, field deployable technique that provides rapid results [5]. Ion mobility spectrometry (IMS) is a desirable chemical analysis technique due to its ease of use, rapid analysis time, and current widespread availability. IMS is a rugged and portable technique that can be used immediately at a crime scene or in theatre to detect contraband substances such as narcotics and explosives [13]. There have been over 10 000 IMS instruments deployed worldwide in airports alone [4]. These screening instruments are used by physically swiping a person’s suitcase, purse, laptop, etc. with a collection wipe to collect trace contaminants. The wipe is then inserted into the instrument and heated to temperatures exceeding 200 °C to thermally desorb the volatile analytes, and after a 7 to 30 second chemical analysis, it produces an indication as to whether explosives (and/or narcotics) were detected. IMS is commonly used in situations (e.g., prison and border checkpoints and airports) requiring high throughput screening for narcotics and/or explosives. One caveat to this method is the need for thermally stable samples that have low chemical background. These potential issues are overcome with this new fingerprint lifting method by using a thermally stable substrate and adhesive with very low chemical background for lifting latent fingerprints.

A typical method for lifting latent fingerprints uses fingerprint powders and inexpensive transparent lifting tape to lift the developed fingerprint. However such fingerprint lifting tape is not suitable for high temperature chemical analyses, as the tape and adhesive would melt during the heating/desorption process and cause significant background interferences in the chemical analyzers. Other chemical analysis techniques such as gas chromatography or liquid chromatography combined with mass spectrometry (GC/MS or LC/MS) could be used after biometric analysis, but these and similar techniques require the dissolution or destruction of the sample in order to perform the chemical analysis, and destroying such evidence is not desirable. With the fingerprint lifting method described here, a latent fingerprint is visualized (i.e., with fingerprint powders) and lifted, and then screened for explosives or narcotics with IMS. The analyzed fingerprint stays intact for subsequent imaging and matching algorithms, typically done at a later time. This fingerprint lifting technique uses a thermally stable substrate and adhesive so that the issues mentioned above are eliminated, providing an opportunity to chemically analyze the lifted fingerprint immediately at a crime scene or later in a laboratory.

## Materials and methods

Experiments were conducted using this method to determine the feasibility of lifting a fingerprint, analyzing it for explosives, and determining the usefulness in a print matching system. A white 0.015 in thick (0.38 mm) Teflon® sheet (McMaster Carr, Chicago, IL) was cut into 1 in × 3 in pieces (25.4 mm × 76.2 mm). A heat resistant, low outgassing silicone adhesive type CV-1161 from NuSil®^1^ (Carpinteria, CA) was diluted with a volume fraction of 1:2 in ethyl acetate solvent (Sigma-Aldrich, St. Louis, MO) and applied to the region of interest on the polytetrafluoroethylene (PTFE, or Teflon®) strips by airbrushing (Aztek Airbrush set, amazon.com). The adhesive coated strips were heated to 230 °C for 1 h to cure, and then were ready to use.

**Fig. 1:**
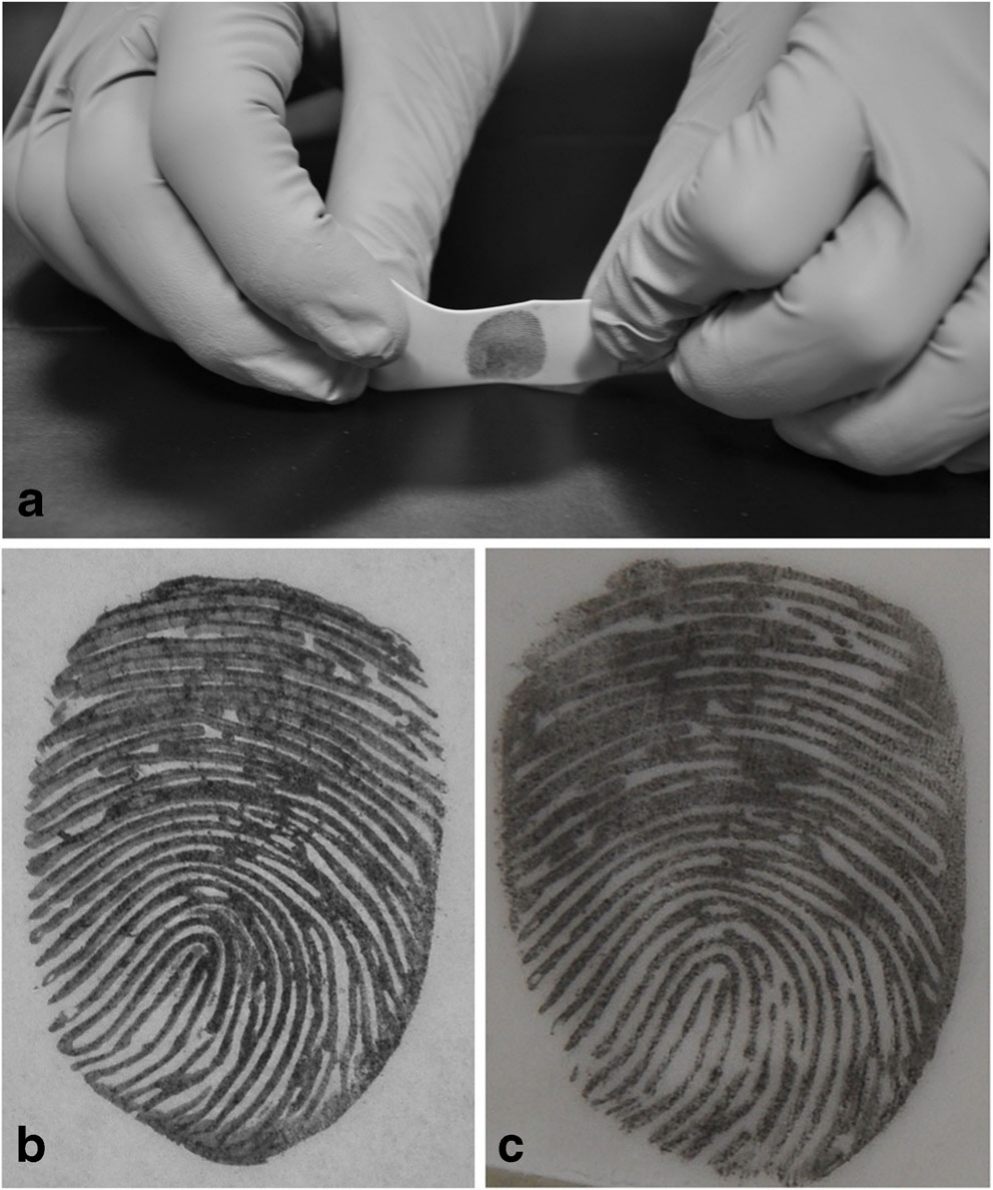
Photographs of lifting fingerprints (These images were made using an artificial fingerprint to protect personally identifiable information (PII). The print was manufactured using computer aided design (CAD) software and fabricated with a 3D rapid prototyping printer. A cast was made of the resulting fake ‘finger’ using dental casting stone, and ballistics gelatin was poured into the cast to create an artificial finger. This gelatin finger was used to deposit sebaceous fingerprints for photographing and publishing purposes. More details of this process will be published elsewhere.). **a** Lifting the powdered latent fingerprint. **b** and **c** are side by side comparisons of resulting fingerprint lifts from a common tape pull using forensic tape (**b**) and the new adhesive swab lift (**c**). Note that (**c**) was originally a mirror image of (**b**); thus computer software was used to horizontally invert the image

Latent fingerprints were made by an anonymous volunteer who pressed their fingers onto clean glass slides. The latent prints were then brushed with black or magnetic fingerprint powder for development. Several additional latent prints containing trace amounts of cyclotrimethylenetrinitramine (RDX) explosive were also prepared using modeling clay containing small amounts of RDX explosive to simulate composition 4 (C-4) plastic explosive. All latent prints were lifted with the prepared fingerprint lifting substrate (Fig. 1). The lifted prints were then scanned at 1000 dpi to create a digital image, and organized in a computer gallery of ‘unknown’ samples, or probes. Known exemplar fingerprints from the same unnamed volunteer were collected on five FD-258 standard fingerprint cards using ink. These cards and the lifted samples were scanned using an FBI Appendix F certified scanning station, and the images were cropped and organized in a gallery of known prints. All images were cropped of most white-space, and the latent fingerprints were inverted across the vertical axis to correct for the inversion resulting from the lift-capture. The digital fingerprints were measured for relative quality using the NIST Fingerprint Image Quality (NFIQ) algorithm [11], processed through the MINDTCT minutiae detector (NIST Biometric Image Software [9]) and the resulting minutiae templates were matched using the BOZORTH3 matcher (NIST Biometric Image Software [9]) to verify that a match can be made between the latent fingerprint and the matching exemplar fingerprint. Twenty samples were compared to 60 known gallery images, for a total of 1200 comparisons. Once a match score for all 20 samples was generated, the samples were analyzed using a 400B IMS instrument (Smith’s Detection, Danbury, CT) or an Itemiser DX IMS instrument (Morpho Detection, Wilmington, MA) where each sample was heated to 230 °C for 7 s. The IMS responses were recorded, and the samples were then rescanned and passed through the matching system again to determine the effect of the heating process on the lifted fingerprints.

## Results and discussion

There are two ways that the performance of the lift medium can be measured. The first is using a monolithic measure tool which can predict/estimate the probability of successfully using the given fingerprint image for matching purposes which is typically referred to as a ‘fingerprint quality’ metric. The second method is to actually scan and match the lifted prints to its known exemplar and obtaining a match score for the image pair. The second method was used here for the collection of experimental data consisting of actual match scores rather than predictive quality estimates. Results have been divided into three basic outcomes; neutral cases, desirable cases, and undesirable cases. A neutral case describes the matching results for comparisons between lifted and exemplar images that remain the same before and after undergoing chemical analysis using IMS. For example, if a fingerprint lift was falsely matched to a known print both before and after chemical analysis, no changes were observed as a result of chemical analysis and therefore it was considered a neutral case. In the traditional sense of quantifying biometric matcher behavior one would consider a false positive match to be a poor result; however, the point of this study was not to test how well the matching algorithm works, but to test whether the chemical analysis affects the matching result. Most of the matching tests (*n* = 1144, > 95 %) resulted as a neutral case because the outcome of the latent fingerprints through the matching system remained unchanged when compared before and after the chemical analysis.

A match case was considered desirable when a false match before chemical analysis became a true rejection after chemical analysis, or when a missed match before chemical analysis became a true match after a chemical analysis. In this situation, the chemical analysis process unintentionally enhanced the lifted fingerprint impression enough to change it from an incorrect answer prior to chemical analysis to a correct answer after chemical analysis in terms of matching results. Three percent of all the matched samples had this desirable outcome. This is considered desirable because we can potentially use the chemical analysis technique to enhance the fingerprint as an aid for matching. It is hypothesized that the sebum in the fingerprint ridges melts slightly, causing the particulates from the fingerprint powder to adhere more strongly to the ridges. An undesirable result is just the opposite; when a correct match result becomes an incorrect match result after the chemical analysis. Less than 2 % of all the analyses had an undesirable result. Table 1 lists a summary of this data.

**Table 1:** Overall probe-gallery fingerprint matches before and after chemical analysis, organized by neutral, desirable, and undesirable results

Condition (before and after IMS)	Count	Match scores (Median)^a^		2-Tailed wilcoxon
		Before heating	After heating	
True Match --> True Match (neutral)	47	41	39	*p* = 0.3224
Missed Match --> Missed Match (neutral)	55	7	8	*p* = 0.0007
Non-Match --> Non-Match (neutral)	1026	6	6	*p* = 0.0002
False Match --> False Match (neutral)	16	15.5	15	*p* = 0.8999
Total count	1144			
False Match --> True rejection (desirable)	27	14	9	*p* = 0.0001
Missed Match --> True Match after (desirable)	9	9	13	*p* = 0.0039
Total count	36			
True-Rejection --> False Match (undesirable)	12	9	14	*p* = 0.0005
True Match --> Missed Match (undesirable)	8	15.5	9	*p* = 0.0078
Total count	20			
Total count of undesirable cases after IMS:	20			
Total count of desirable/neutral cases after IMS:	1180			

^a^positive match threshold set to score value of 13

**Fig. 2:**
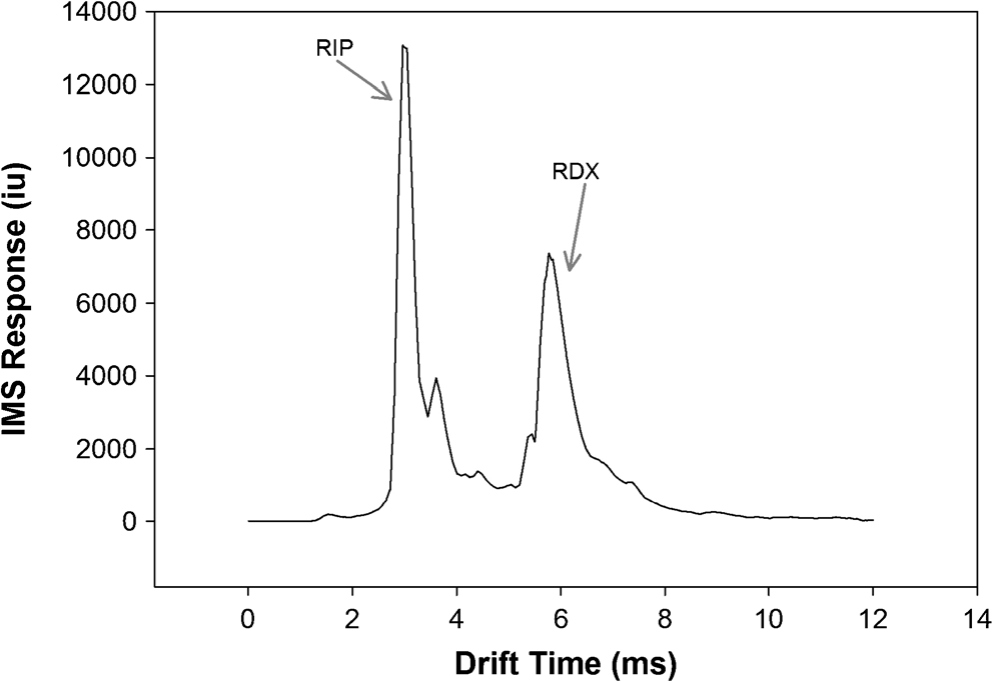
IMS spectrum of an RDX alarm for latent fingerprint lifts. The IMS response is shown as the maximum intensity units (iu) of 120 scans at a given drift time. Notice there are no significant chemical background peaks

A select number of fingerprints were prepared using a simulated plastic bonded explosive. These samples were not used in the match study mainly due to issues of potentially contaminating various laboratory surfaces and the image scanner. These lifted fingerprints with simulated explosive contamination were prepared only for IMS analysis, to ensure that residue left in a latent fingerprint could be detected in a trace contraband detector. In order to avoid handling the explosives, gelatin fingers prepared from dental casting stone as previously described were used. The artificial gelatin fingers were pressed into modeling clay containing small amounts of RDX to simulate composition 4 (C-4) plastic bonded explosive. The RDX contaminated gelatin fingers were then pressed onto clean glass slides. This was a qualitative study because the mass of simulated explosive deposited in each print was not measured or controlled. All 14 prints analyzed produced an IMS response with a relative standard deviation (RSD) of 49.8 %. The results appear to be variable due to the high RSD, but this is because the mass of explosive present in each sample is unknown. This represents a more realistic distribution since fingerprints can contain variable amounts of explosive even when depositing several fingerprints from handling a single piece of explosive [12]. An IMS spectrum of the resulting RDX detection is shown in Fig. 2.

## Conclusions

The results of this study show the feasibility of lifting a latent fingerprint using this novel method and chemically analyzing it immediately without destroying the lifted print. We have shown that the powdered fingerprints lifted with the high-temperature adhesive media are useful in a fingerprint matching system. We have also shown that explosives residues present in such lifted fingerprints can be successfully screened and explosives detected using trace detection equipment.

An application of the described technique would be for military personnel in theatre when they come in contact with a suspicious package that could be an improvised explosive device (IED). They could quickly brush the package for prints, lift the print with the fingerprint lifting media, and analyze it immediately with a field-ready trace explosives detector. The analyzed fingerprint could be saved for subsequent matching to try and determine who has handled the package. Such analysis is not currently available. Future efforts will include adding fiducial marks for easier scanning and matching and finding an ideal protective covering for the lifting medium for both before and after lifting a fingerprint.

## Footnotes

1 Certain commercial equipment, instruments, or materials are identified in this document. Such identification does not imply recommendation or endorsement by the National Institute of Standards and Technology, nor does it imply that the products identified are necessarily the best available for the purpose.
